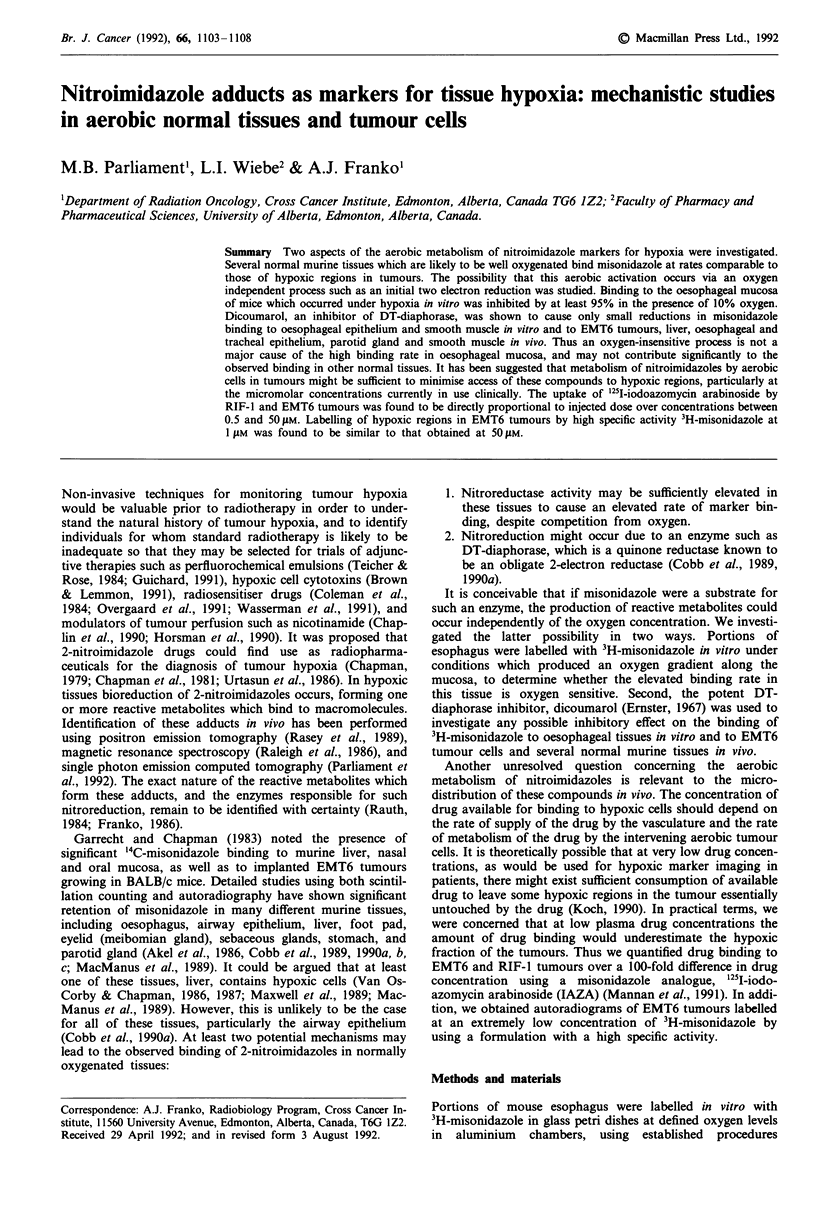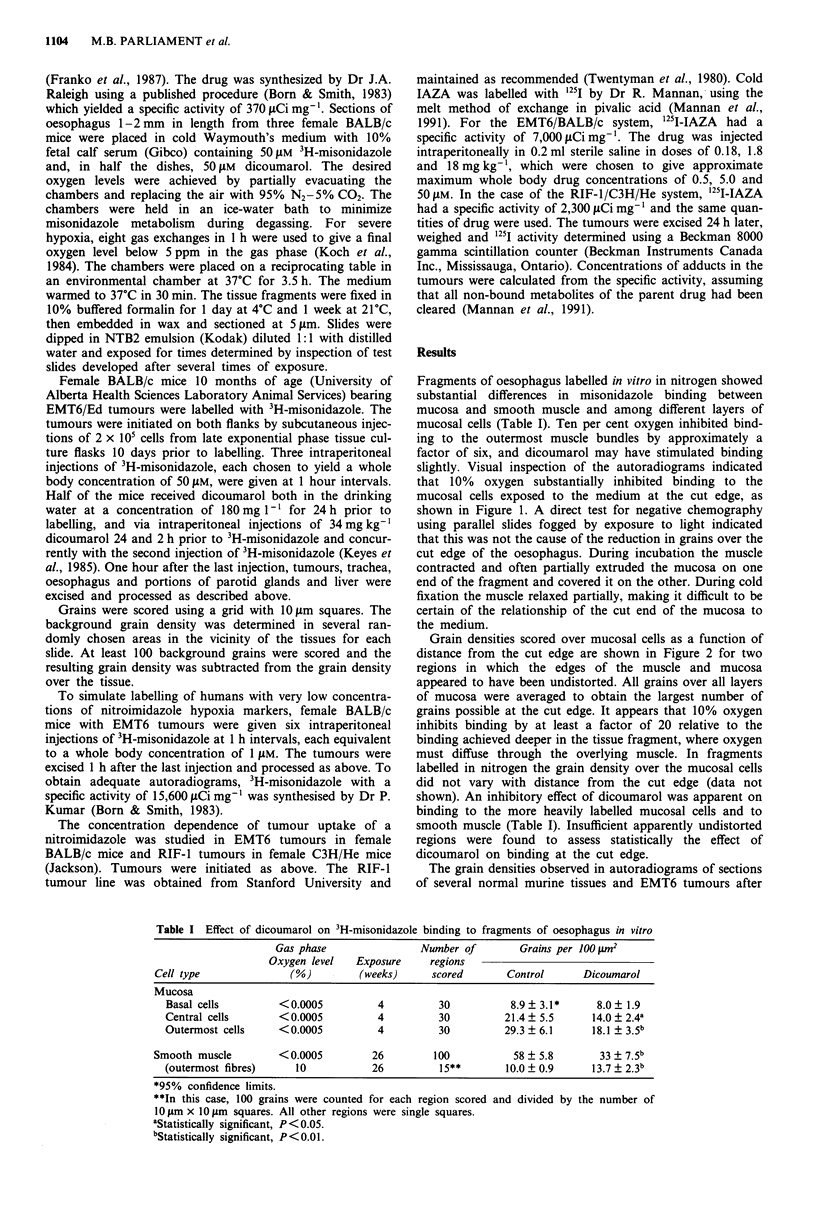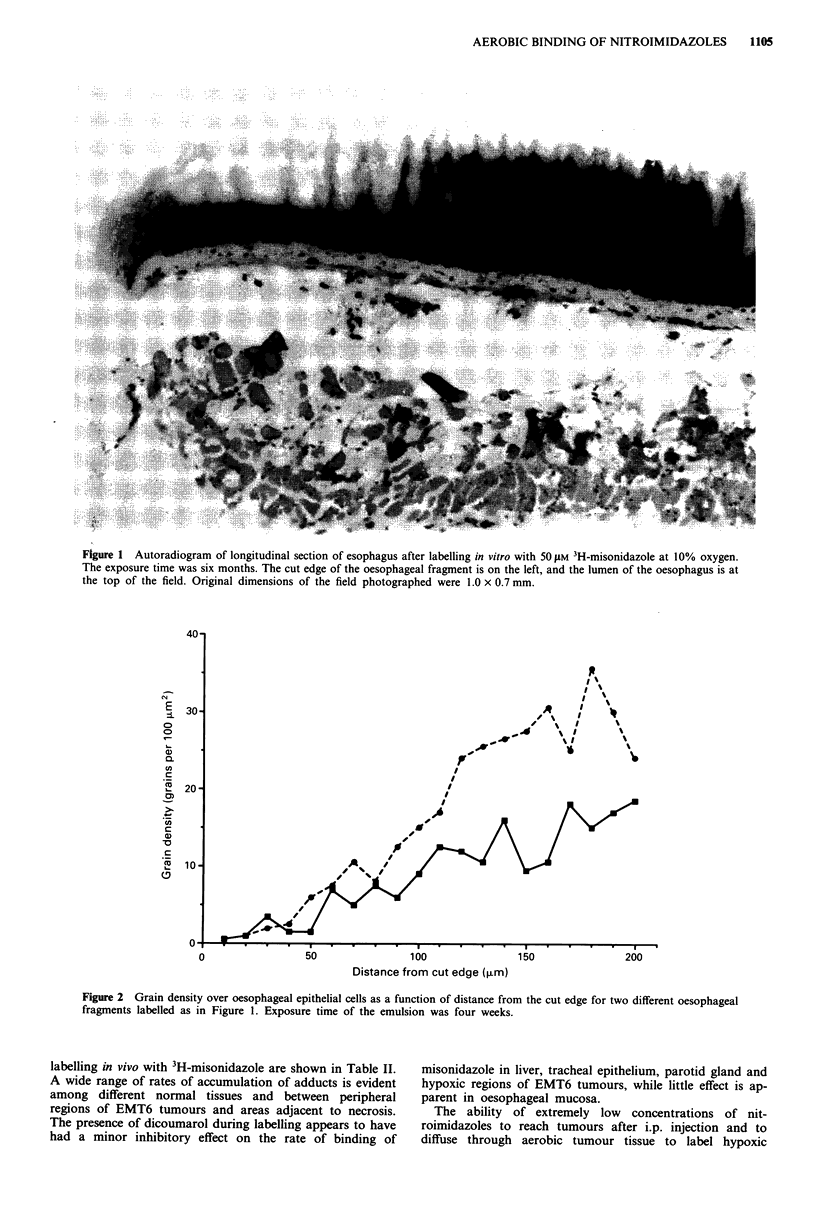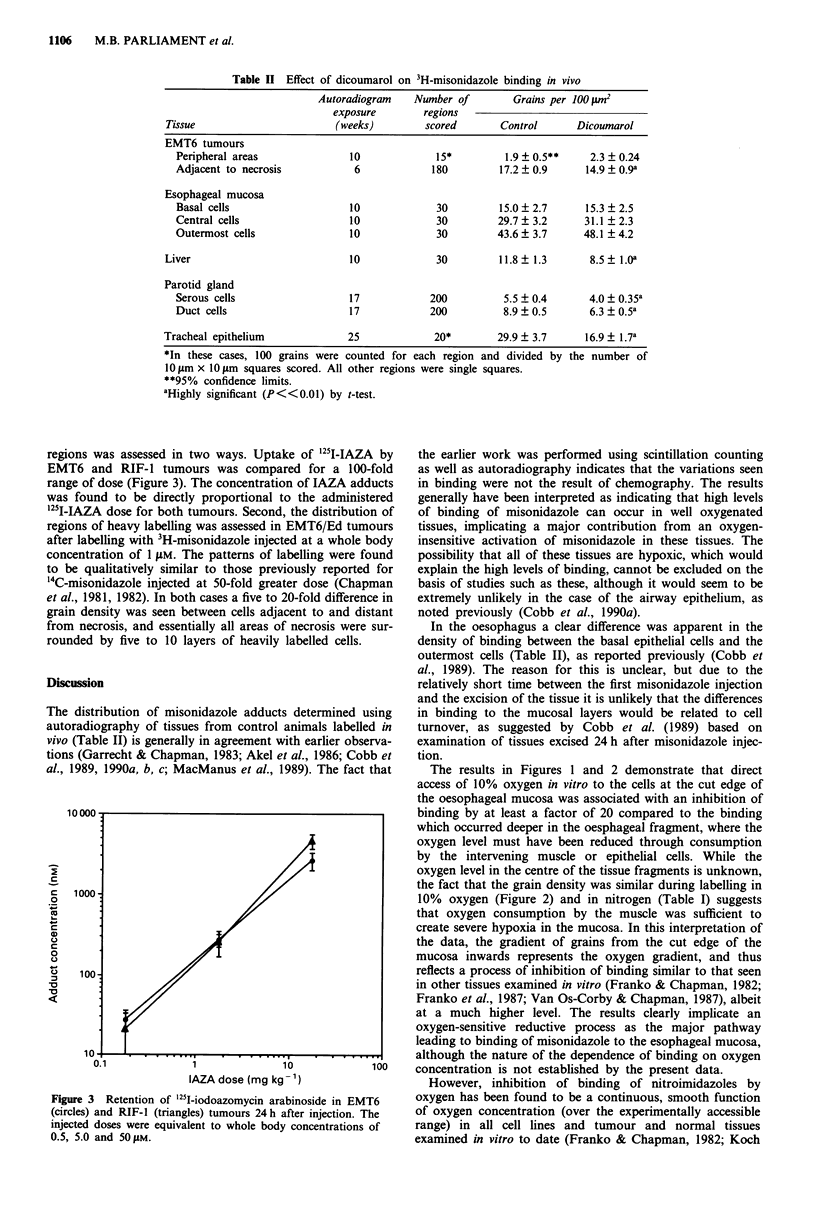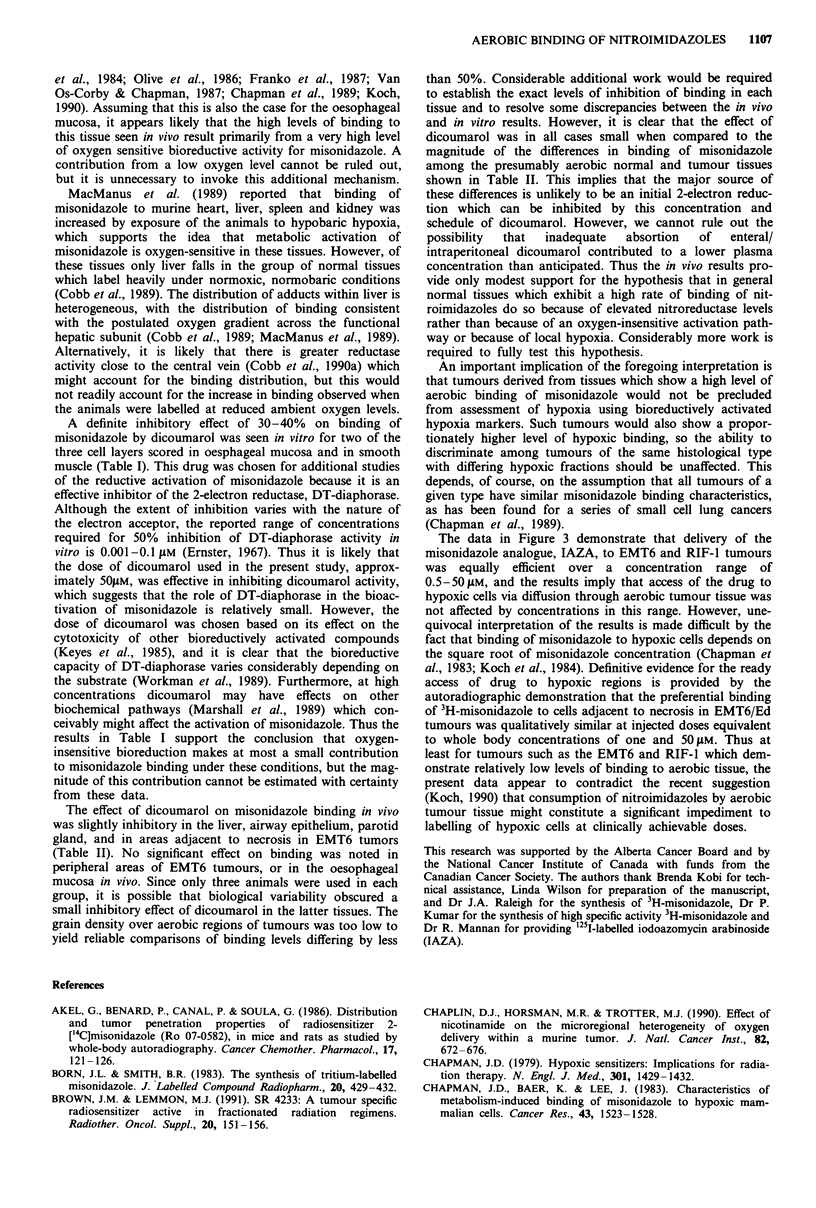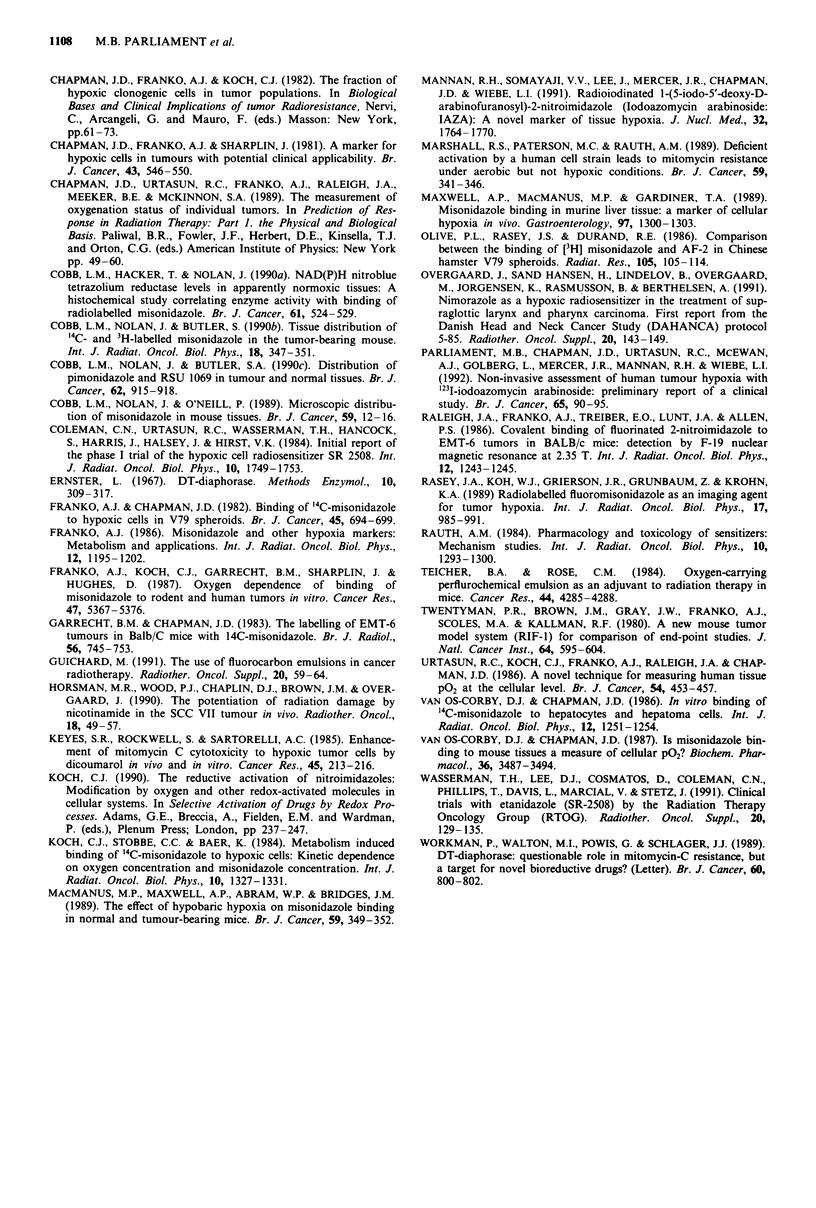# Nitroimidazole adducts as markers for tissue hypoxia: mechanistic studies in aerobic normal tissues and tumour cells.

**DOI:** 10.1038/bjc.1992.418

**Published:** 1992-12

**Authors:** M. B. Parliament, L. I. Wiebe, A. J. Franko

**Affiliations:** Department of Radiation Oncology, Cross Cancer Institute, Edmonton, Alberta, Canada.

## Abstract

**Images:**


Br. J. Cancer (1992), 66, 1103–1108 © Macmillan Press Ltd., 1992

### Nitroimidazole adducts as markers for tissue hypoxia: mechanistic studies in aerobic normal tissues and tumour cells

M.B. Parliament[1], L.I. Wiebe[2] & A.J. Franko[1]

[1]*Department of Radiation Oncology, Cross Cancer Institute, Edmonton, Alberta, Canada TG6 1Z2;* [2]*Faculty of Pharmacy and Pharmaceutical Sciences, University of Alberta, Edmonton, Alberta, Canada.*

**Summary** Two aspects of the aerobic metabolism of nitroimidazole markers for hypoxia were investigated. Several normal murine tissues which are likely to be well oxygenated bind misonidazole at rates comparable to those of hypoxic regions in tumours. The possibility that this aerobic activation occurs via an oxygen independent process such as an initial two electron reduction was studied. Binding to the oesophageal mucosa of mice which occurred under hypoxia *in vitro* was inhibited by at least 95% in the presence of 10% oxygen. Dicoumarol, an inhibitor of DT-diaphorase, was shown to cause only small reductions in misonidazole binding to oesophageal epithelium and smooth muscle *in vitro* and to EMT6 tumours, liver, oesophageal and tracheal epithelium, parotid gland and smooth muscle *in vivo*. Thus an oxygen-insensitive process is not a major cause of the high binding rate in oesophageal mucosa, and may not contribute significantly to the observed binding in other normal tissues. It has been suggested that metabolism of nitroimidazoles by aerobic cells in tumours might be sufficient to minimise access of these compounds to hypoxic regions, particularly at the micromolar concentrations currently in use clinically. The uptake of $^{125}$I-iodoazomycin arabinoside by RIF-1 and EMT6 tumours was found to be directly proportional to injected dose over concentrations between 0.5 and 50 µM. Labelling of hypoxic regions in EMT6 tumours by high specific activity $^{3}$H-misonidazole at 1 µM was found to be similar to that obtained at 50 µM.

Non-invasive techniques for monitoring tumour hypoxia would be valuable prior to radiotherapy in order to understand the natural history of tumour hypoxia, and to identify individuals for whom standard radiotherapy is likely to be inadequate so that they may be selected for trials of adjunctive therapies such as perfluorochemical emulsions (Teicher & Rose, 1984; Guichard, 1991), hypoxic cell cytotoxins (Brown & Lemmon, 1991), radiosensitiser drugs (Coleman *et al.*, 1984; Overgaard *et al.*, 1991; Wasserman *et al.*, 1991), and modulators of tumour perfusion such as nicotinamide (Chaplin *et al.*, 1990; Horsman *et al.*, 1990). It was proposed that 2-nitroimidazole drugs could find use as radiopharmaceuticals for the diagnosis of tumour hypoxia (Chapman, 1979; Chapman *et al.*, 1981; Urtasun *et al.*, 1986). In hypoxic tissues bioreduction of 2-nitroimidazoles occurs, forming one or more reactive metabolites which bind to macromolecules. Identification of these adducts *in vivo* has been performed using positron emission tomography (Rasey *et al.*, 1989), magnetic resonance spectroscopy (Raleigh *et al.*, 1986), and single photon emission computed tomography (Parliament *et al.*, 1992). The exact nature of the reactive metabolites which form these adducts, and the enzymes responsible for such nitroreduction, remain to be identified with certainty (Rauth, 1984; Franko, 1986).

Garrecht and Chapman (1983) noted the presence of significant $^{14}$C-misonidazole binding to murine liver, nasal and oral mucosa, as well as to implanted EMT6 tumours growing in BALB/c mice. Detailed studies using both scintillation counting and autoradiography have shown significant retention of misonidazole in many different murine tissues, including oesophagus, airway epithelium, liver, foot pad, eyelid (meibomian gland), sebaceous glands, stomach, and parotid gland (Akel *et al.*, 1986, Cobb *et al.*, 1989, 1990*a, b, c*; MacManus *et al.*, 1989). It could be argued that at least one of these tissues, liver, contains hypoxic cells (Van Os-Corby & Chapman, 1986, 1987; Maxwell *et al.*, 1989; MacManus *et al.*, 1989). However, this is unlikely to be the case for all of these tissues, particularly the airway epithelium (Cobb *et al.*, 1990*a*). At least two potential mechanisms may lead to the observed binding of 2-nitroimidazoles in normally oxygenated tissues:

1. Nitroreductase activity may be sufficiently elevated in these tissues to cause an elevated rate of marker binding, despite competition from oxygen.
2. Nitroreduction might occur due to an enzyme such as DT-diaphorase, which is a quinone reductase known to be an obligate 2-electron reductase (Cobb *et al.*, 1989, 1990*a*).

It is conceivable that if misonidazole were a substrate for such an enzyme, the production of reactive metabolites could occur independently of the oxygen concentration. We investigated the latter possibility in two ways. Portions of esophagus were labelled with $^{3}$H-misonidazole *in vitro* under conditions which produced an oxygen gradient along the mucosa, to determine whether the elevated binding rate in this tissue is oxygen sensitive. Second, the potent DT-diaphorase inhibitor, dicoumarol (Ernster, 1967) was used to investigate any possible inhibitory effect on the binding of $^{3}$H-misonidazole to oesophageal tissues *in vitro* and to EMT6 tumour cells and several normal murine tissues *in vivo*.

Another unresolved question concerning the aerobic metabolism of nitroimidazoles is relevant to the microdistribution of these compounds *in vivo*. The concentration of drug available for binding to hypoxic cells should depend on the rate of supply of the drug by the vasculature and the rate of metabolism of the drug by the intervening aerobic tumour cells. It is theoretically possible that at very low drug concentrations, as would be used for hypoxic marker imaging in patients, there might exist sufficient consumption of available drug to leave some hypoxic regions in the tumour essentially untouched by the drug (Koch, 1990). In practical terms, we were concerned that at low plasma drug concentrations the amount of drug binding would underestimate the hypoxic fraction of the tumours. Thus we quantified drug binding to EMT6 and RIF-1 tumours over a 100-fold difference in drug concentration using a misonidazole analogue, $^{125}$I-iodoazomycin arabinoside (IAZA) (Mannan *et al.*, 1991). In addition, we obtained autoradiograms of EMT6 tumours labelled at an extremely low concentration of $^{3}$H-misonidazole by using a formulation with a high specific activity.

### Methods and materials

Portions of mouse esophagus were labelled *in vitro* with $^{3}$H-misonidazole in glass petri dishes at defined oxygen levels in aluminium chambers, using established procedures

Correspondence: A.J. Franko, Radiobiology Program, Cross Cancer Institute, 11560 University Avenue, Edmonton, Alberta, Canada, T6G 1Z2.
Received 29 April 1992; and in revised form 3 August 1992.

(Franko *et al.*, 1987). The drug was synthesized by Dr J.A. Raleigh using a published procedure (Born & Smith, 1983) which yielded a specific activity of 370 μCi $mg^{-1}$. Sections of oesophagus 1–2 mm in length from three female BALB/c mice were placed in cold Waymouth's medium with 10% fetal calf serum (Gibco) containing 50 μM $^3$H-misonidazole and, in half the dishes, 50 μM dicoumarol. The desired oxygen levels were achieved by partially evacuating the chambers and replacing the air with 95% $N_2$–5% $CO_2$. The chambers were held in an ice-water bath to minimize misonidazole metabolism during degassing. For severe hypoxia, eight gas exchanges in 1 h were used to give a final oxygen level below 5 ppm in the gas phase (Koch *et al.*, 1984). The chambers were placed on a reciprocating table in an environmental chamber at 37°C for 3.5 h. The medium warmed to 37°C in 30 min. The tissue fragments were fixed in 10% buffered formalin for 1 day at 4°C and 1 week at 21°C, then embedded in wax and sectioned at 5 μm. Slides were dipped in NTB2 emulsion (Kodak) diluted 1:1 with distilled water and exposed for times determined by inspection of test slides developed after several times of exposure.

Female BALB/c mice 10 months of age (University of Alberta Health Sciences Laboratory Animal Services) bearing EMT6/Ed tumours were labelled with $^3$H-misonidazole. The tumours were initiated on both flanks by subcutaneous injections of $2 \times 10^5$ cells from late exponential phase tissue culture flasks 10 days prior to labelling. Three intraperitoneal injections of $^3$H-misonidazole, each chosen to yield a whole body concentration of 50 μM, were given at 1 hour intervals. Half of the mice received dicoumarol both in the drinking water at a concentration of 180 mg $l^{-1}$ for 24 h prior to labelling, and via intraperitoneal injections of 34 mg $kg^{-1}$ dicoumarol 24 and 2 h prior to $^3$H-misonidazole and concurrently with the second injection of $^3$H-misonidazole (Keyes *et al.*, 1985). One hour after the last injection, tumours, trachea, oesophagus and portions of parotid glands and liver were excised and processed as described above.

Grains were scored using a grid with 10 μm squares. The background grain density was determined in several randomly chosen areas in the vicinity of the tissues for each slide. At least 100 background grains were scored and the resulting grain density was subtracted from the grain density over the tissue.

To simulate labelling of humans with very low concentrations of nitroimidazole hypoxia markers, female BALB/c mice with EMT6 tumours were given six intraperitoneal injections of $^3$H-misonidazole at 1 h intervals, each equivalent to a whole body concentration of 1 μM. The tumours were excised 1 h after the last injection and processed as above. To obtain adequate autoradiograms, $^3$H-misonidazole with a specific activity of 15,600 μCi $mg^{-1}$ was synthesised by Dr P. Kumar (Born & Smith, 1983).

The concentration dependence of tumour uptake of a nitroimidazole was studied in EMT6 tumours in female BALB/c mice and RIF-1 tumours in female C3H/He mice (Jackson). Tumours were initiated as above. The RIF-1 tumour line was obtained from Stanford University and maintained as recommended (Twentyman *et al.*, 1980). Cold IAZA was labelled with $^{125}$I by Dr R. Mannan, using the melt method of exchange in pivalic acid (Mannan *et al.*, 1991). For the EMT6/BALB/c system, $^{125}$I-IAZA had a specific activity of 7,000 μCi $mg^{-1}$. The drug was injected intraperitoneally in 0.2 ml sterile saline in doses of 0.18, 1.8 and 18 mg $kg^{-1}$, which were chosen to give approximate maximum whole body drug concentrations of 0.5, 5.0 and 50 μM. In the case of the RIF-1/C3H/He system, $^{125}$I-IAZA had a specific activity of 2,300 μCi $mg^{-1}$ and the same quantities of drug were used. The tumours were excised 24 h later, weighed and $^{125}$I activity determined using a Beckman 8000 gamma scintillation counter (Beckman Instruments Canada Inc., Mississauga, Ontario). Concentrations of adducts in the tumours were calculated from the specific activity, assuming that all non-bound metabolites of the parent drug had been cleared (Mannan *et al.*, 1991).

### Results

Fragments of oesophagus labelled *in vitro* in nitrogen showed substantial differences in misonidazole binding between mucosa and smooth muscle and among different layers of mucosal cells (Table I). Ten per cent oxygen inhibited binding to the outermost muscle bundles by approximately a factor of six, and dicoumarol may have stimulated binding slightly. Visual inspection of the autoradiograms indicated that 10% oxygen substantially inhibited binding to the mucosal cells exposed to the medium at the cut edge, as shown in Figure 1. A direct test for negative chemography using parallel slides fogged by exposure to light indicated that this was not the cause of the reduction in grains over the cut edge of the oesophagus. During incubation the muscle contracted and often partially extruded the mucosa on one end of the fragment and covered it on the other. During cold fixation the muscle relaxed partially, making it difficult to be certain of the relationship of the cut end of the mucosa to the medium.

Grain densities scored over mucosal cells as a function of distance from the cut edge are shown in Figure 2 for two regions in which the edges of the muscle and mucosa appeared to have been undistorted. All grains over all layers of mucosa were averaged to obtain the largest number of grains possible at the cut edge. It appears that 10% oxygen inhibits binding by at least a factor of 20 relative to the binding achieved deeper in the tissue fragment, where oxygen must diffuse through the overlying muscle. In fragments labelled in nitrogen the grain density over the mucosal cells did not vary with distance from the cut edge (data not shown). An inhibitory effect of dicoumarol was apparent on binding to the more heavily labelled mucosal cells and to smooth muscle (Table I). Insufficient apparently undistorted regions were found to assess statistically the effect of dicoumarol on binding at the cut edge.

The grain densities observed in autoradiograms of sections of several normal murine tissues and EMT6 tumours after

**Table I** Effect of dicoumarol on $^3$H-misonidazole binding to fragments of oesophagus *in vitro*

| Cell type | *Gas phase Oxygen level (%)* | *Exposure (weeks)* | *Number of regions scored* | *Grains per 100 μm²* | |
|---|---|---|---|---|---|
| | | | | *Control* | *Dicoumarol* |
| Mucosa | | | | | |
| Basal cells | <0.0005 | 4 | 30 | 8.9 ± 3.1* | 8.0 ± 1.9 |
| Central cells | <0.0005 | 4 | 30 | 21.4 ± 5.5 | 14.0 ± 2.4[a] |
| Outermost cells | <0.0005 | 4 | 30 | 29.3 ± 6.1 | 18.1 ± 3.5[b] |
| Smooth muscle | <0.0005 | 26 | 100 | 58 ± 5.8 | 33 ± 7.5[b] |
| (outermost fibres) | 10 | 26 | 15** | 10.0 ± 0.9 | 13.7 ± 2.3[b] |

*95% confidence limits.
**In this case, 100 grains were counted for each region scored and divided by the number of 10 μm × 10 μm squares. All other regions were single squares.
[a]Statistically significant, $P < 0.05$.
[b]Statistically significant, $P < 0.01$.

**Figure 1** Autoradiogram of longitudinal section of esophagus after labelling *in vitro* with 50 μM $^3$H-misonidazole at 10% oxygen. The exposure time was six months. The cut edge of the oesophageal fragment is on the left, and the lumen of the oesophagus is at the top of the field. Original dimensions of the field photographed were 1.0 × 0.7 mm.

**Figure 2** Grain density over oesophageal epithelial cells as a function of distance from the cut edge for two different oesophageal fragments labelled as in Figure 1. Exposure time of the emulsion was four weeks.

labelling *in vivo* with $^3$H-misonidazole are shown in Table II. A wide range of rates of accumulation of adducts is evident among different normal tissues and between peripheral regions of EMT6 tumours and areas adjacent to necrosis. The presence of dicoumarol during labelling appears to have had a minor inhibitory effect on the rate of binding of misonidazole in liver, tracheal epithelium, parotid gland and hypoxic regions of EMT6 tumours, while little effect is apparent in oesophageal mucosa.

The ability of extremely low concentrations of nitroimidazoles to reach tumours after i.p. injection and to diffuse through aerobic tumour tissue to label hypoxic

**Table II** Effect of dicoumarol on $^{3}$H-misonidazole binding *in vivo*

| *Tissue* | *Autoradiogram exposure (weeks)* | *Number of regions scored* | *Grains per 100 μm²* | |
|---|---|---|---|---|
| | | | *Control* | *Dicoumarol* |
| EMT6 tumours | | | | |
| Peripheral areas | 10 | 15* | 1.9 ± 0.5** | 2.3 ± 0.24 |
| Adjacent to necrosis | 6 | 180 | 17.2 ± 0.9 | 14.9 ± 0.9[a] |
| Esophageal mucosa | | | | |
| Basal cells | 10 | 30 | 15.0 ± 2.7 | 15.3 ± 2.5 |
| Central cells | 10 | 30 | 29.7 ± 3.2 | 31.1 ± 2.3 |
| Outermost cells | 10 | 30 | 43.6 ± 3.7 | 48.1 ± 4.2 |
| Liver | 10 | 30 | 11.8 ± 1.3 | 8.5 ± 1.0[a] |
| Parotid gland | | | | |
| Serous cells | 17 | 200 | 5.5 ± 0.4 | 4.0 ± 0.35[a] |
| Duct cells | 17 | 200 | 8.9 ± 0.5 | 6.3 ± 0.5[a] |
| Tracheal epithelium | 25 | 20* | 29.9 ± 3.7 | 16.9 ± 1.7[a] |

*In these cases, 100 grains were counted for each region and divided by the number of 10 μm × 10 μm squares scored. All other regions were single squares.
**95% confidence limits.
[a]Highly significant ($P << 0.01$) by $t$-test.

regions was assessed in two ways. Uptake of $^{125}$I-IAZA by EMT6 and RIF-1 tumours was compared for a 100-fold range of dose (Figure 3). The concentration of IAZA adducts was found to be directly proportional to the administered $^{125}$I-IAZA dose for both tumours. Second, the distribution of regions of heavy labelling was assessed in EMT6/Ed tumours after labelling with $^{3}$H-misonidazole injected at a whole body concentration of 1 μM. The patterns of labelling were found to be qualitatively similar to those previously reported for $^{14}$C-misonidazole injected at 50-fold greater dose (Chapman *et al.*, 1981, 1982). In both cases a five to 20-fold difference in grain density was seen between cells adjacent to and distant from necrosis, and essentially all areas of necrosis were surrounded by five to 10 layers of heavily labelled cells.

### Discussion

The distribution of misonidazole adducts determined using autoradiography of tissues from control animals labelled *in vivo* (Table II) is generally in agreement with earlier observations (Garrecht & Chapman, 1983; Akel *et al.*, 1986; Cobb *et al.*, 1989, 1990*a, b, c*; MacManus *et al.*, 1989). The fact that

**Figure 3** Retention of $^{125}$I-iodoazomycin arabinoside in EMT6 (circles) and RIF-1 (triangles) tumours 24 h after injection. The injected doses were equivalent to whole body concentrations of 0.5, 5.0 and 50 μM.

the earlier work was performed using scintillation counting as well as autoradiography indicates that the variations seen in binding were not the result of chemography. The results generally have been interpreted as indicating that high levels of binding of misonidazole can occur in well oxygenated tissues, implicating a major contribution from an oxygen-insensitive activation of misonidazole in these tissues. The possibility that all of these tissues are hypoxic, which would explain the high levels of binding, cannot be excluded on the basis of studies such as these, although it would seem to be extremely unlikely in the case of the airway epithelium, as noted previously (Cobb *et al.*, 1990*a*).

In the oesophagus a clear difference was apparent in the density of binding between the basal epithelial cells and the outermost cells (Table II), as reported previously (Cobb *et al.*, 1989). The reason for this is unclear, but due to the relatively short time between the first misonidazole injection and the excision of the tissue it is unlikely that the differences in binding to the mucosal layers would be related to cell turnover, as suggested by Cobb *et al.* (1989) based on examination of tissues excised 24 h after misonidazole injection.

The results in Figures 1 and 2 demonstrate that direct access of 10% oxygen *in vitro* to the cells at the cut edge of the oesophageal mucosa was associated with an inhibition of binding by at least a factor of 20 compared to the binding which occurred deeper in the oesphageal fragment, where the oxygen level must have been reduced through consumption by the intervening muscle or epithelial cells. While the oxygen level in the centre of the tissue fragments is unknown, the fact that the grain density was similar during labelling in 10% oxygen (Figure 2) and in nitrogen (Table I) suggests that oxygen consumption by the muscle was sufficient to create severe hypoxia in the mucosa. In this interpretation of the data, the gradient of grains from the cut edge of the mucosa inwards represents the oxygen gradient, and thus reflects a process of inhibition of binding similar to that seen in other tissues examined *in vitro* (Franko & Chapman, 1982; Franko *et al.*, 1987; Van Os-Corby & Chapman, 1987), albeit at a much higher level. The results clearly implicate an oxygen-sensitive reductive process as the major pathway leading to binding of misonidazole to the esophageal mucosa, although the nature of the dependence of binding on oxygen concentration is not established by the present data.

However, inhibition of binding of nitroimidazoles by oxygen has been found to be a continuous, smooth function of oxygen concentration (over the experimentally accessible range) in all cell lines and tumour and normal tissues examined *in vitro* to date (Franko & Chapman, 1982; Koch et al., 1984; Olive et al., 1986; Franko et al., 1987; Van Os-Corby & Chapman, 1987; Chapman et al., 1989; Koch, 1990). Assuming that this is also the case for the oesophageal mucosa, it appears likely that the high levels of binding to this tissue seen *in vivo* result primarily from a very high level of oxygen sensitive bioreductive activity for misonidazole. A contribution from a low oxygen level cannot be ruled out, but it is unnecessary to invoke this additional mechanism.

MacManus et al. (1989) reported that binding of misonidazole to murine heart, liver, spleen and kidney was increased by exposure of the animals to hypobaric hypoxia, which supports the idea that metabolic activation of misonidazole is oxygen-sensitive in these tissues. However, of these tissues only liver falls in the group of normal tissues which label heavily under normoxic, normobaric conditions (Cobb et al., 1989). The distribution of adducts within liver is heterogeneous, with the distribution of binding consistent with the postulated oxygen gradient across the functional hepatic subunit (Cobb et al., 1989; MacManus et al., 1989). Alternatively, it is likely that there is greater reductase activity close to the central vein (Cobb et al., 1990a) which might account for the binding distribution, but this would not readily account for the increase in binding observed when the animals were labelled at reduced ambient oxygen levels.

A definite inhibitory effect of 30–40% on binding of misonidazole by dicoumarol was seen *in vitro* for two of the three cell layers scored in oesphageal mucosa and in smooth muscle (Table I). This drug was chosen for additional studies of the reductive activation of misonidazole because it is an effective inhibitor of the 2-electron reductase, DT-diaphorase. Although the extent of inhibition varies with the nature of the electron acceptor, the reported range of concentrations required for 50% inhibition of DT-diaphorase activity *in vitro* is 0.001–0.1 μM (Ernster, 1967). Thus it is likely that the dose of dicoumarol used in the present study, approximately 50μM, was effective in inhibiting dicoumarol activity, which suggests that the role of DT-diaphorase in the bioactivation of misonidazole is relatively small. However, the dose of dicoumarol was chosen based on its effect on the cytotoxicity of other bioreductively activated compounds (Keyes et al., 1985), and it is clear that the bioreductive capacity of DT-diaphorase varies considerably depending on the substrate (Workman et al., 1989). Furthermore, at high concentrations dicoumarol may have effects on other biochemical pathways (Marshall et al., 1989) which conceivably might affect the activation of misonidazole. Thus the results in Table I support the conclusion that oxygen-insensitive bioreduction makes at most a small contribution to misonidazole binding under these conditions, but the magnitude of this contribution cannot be estimated with certainty from these data.

The effect of dicoumarol on misonidazole binding *in vivo* was slightly inhibitory in the liver, airway epithelium, parotid gland, and in areas adjacent to necrosis in EMT6 tumors (Table II). No significant effect on binding was noted in peripheral areas of EMT6 tumours, or in the oesophageal mucosa *in vivo*. Since only three animals were used in each group, it is possible that biological variability obscured a small inhibitory effect of dicoumarol in the latter tissues. The grain density over aerobic regions of tumours was too low to yield reliable comparisons of binding levels differing by less than 50%. Considerable additional work would be required to establish the exact levels of inhibition of binding in each tissue and to resolve some discrepancies between the *in vivo* and *in vitro* results. However, it is clear that the effect of dicoumarol was in all cases small when compared to the magnitude of the differences in binding of misonidazole among the presumably aerobic normal and tumour tissues shown in Table II. This implies that the major source of these differences is unlikely to be an initial 2-electron reduction which can be inhibited by this concentration and schedule of dicoumarol. However, we cannot rule out the possibility that inadequate absorption of enteral/intraperitoneal dicoumarol contributed to a lower plasma concentration than anticipated. Thus the *in vivo* results provide only modest support for the hypothesis that in general normal tissues which exhibit a high rate of binding of nitroimidazoles do so because of elevated nitroreductase levels rather than because of an oxygen-insensitive activation pathway or because of local hypoxia. Considerably more work is required to fully test this hypothesis.

An important implication of the foregoing interpretation is that tumours derived from tissues which show a high level of aerobic binding of misonidazole would not be precluded from assessment of hypoxia using bioreductively activated hypoxia markers. Such tumours would also show a proportionately higher level of hypoxic binding, so the ability to discriminate among tumours of the same histological type with differing hypoxic fractions should be unaffected. This depends, of course, on the assumption that all tumours of a given type have similar misonidazole binding characteristics, as has been found for a series of small cell lung cancers (Chapman et al., 1989).

The data in Figure 3 demonstrate that delivery of the misonidazole analogue, IAZA, to EMT6 and RIF-1 tumours was equally efficient over a concentration range of 0.5–50 μM, and the results imply that access of the drug to hypoxic cells via diffusion through aerobic tumour tissue was not affected by concentrations in this range. However, unequivocal interpretation of the results is made difficult by the fact that binding of misonidazole to hypoxic cells depends on the square root of misonidazole concentration (Chapman et al., 1983; Koch et al., 1984). Definitive evidence for the ready access of drug to hypoxic regions is provided by the autoradiographic demonstration that the preferential binding of $^{3}$H-misonidazole to cells adjacent to necrosis in EMT6/Ed tumours was qualitatively similar at injected doses equivalent to whole body concentrations of one and 50 μM. Thus at least for tumours such as the EMT6 and RIF-1 which demonstrate relatively low levels of binding to aerobic tissue, the present data appear to contradict the recent suggestion (Koch, 1990) that consumption of nitroimidazoles by aerobic tumour tissue might constitute a significant impediment to labelling of hypoxic cells at clinically achievable doses.

This research was supported by the Alberta Cancer Board and by the National Cancer Institute of Canada with funds from the Canadian Cancer Society. The authors thank Brenda Kobi for technical assistance, Linda Wilson for preparation of the manuscript, and Dr J.A. Raleigh for the synthesis of $^{3}$H-misonidazole, Dr P. Kumar for the synthesis of high specific activity $^{3}$H-misonidazole and Dr R. Mannan for providing $^{125}$I-labelled iodoazomycin arabinoside (IAZA).

### References

AKEL, G., BENARD, P., CANAL, P. & SOULA, G. (1986). Distribution and tumor penetration properties of radiosensitizer 2-[$^{14}$C]misonidazole (Ro 07-0582), in mice and rats as studied by whole-body autoradiography. *Cancer Chemother. Pharmacol.*, **17**, 121–126.

BORN, J.L. & SMITH, B.R. (1983). The synthesis of tritium-labelled misonidazole. *J. Labelled Compound Radiopharm.*, **20**, 429–432.

BROWN, J.M. & LEMMON, M.J. (1991). SR 4233: A tumour specific radiosensitizer active in fractionated radiation regimens. *Radiother. Oncol. Suppl.*, **20**, 151–156.

CHAPLIN, D.J., HORSMAN, M.R. & TROTTER, M.J. (1990). Effect of nicotinamide on the microregional heterogeneity of oxygen delivery within a murine tumor. *J. Natl. Cancer Inst.*, **82**, 672–676.

CHAPMAN, J.D. (1979). Hypoxic sensitizers: Implications for radiation therapy. *N. Engl. J. Med.*, **301**, 1429–1432.

CHAPMAN, J.D., BAER, K. & LEE, J. (1983). Characteristics of metabolism-induced binding of misonidazole to hypoxic mammalian cells. *Cancer Res.*, **43**, 1523–1528.

CHAPMAN, J.D., FRANKO, A.J. & KOCH, C.J. (1982). The fraction of hypoxic clonogenic cells in tumor populations. In *Biological Bases and Clinical Implications of tumor Radioresistance*, Nervi, C., Arcangeli, G. and Mauro, F. (eds.) Masson: New York, pp.61-73.

CHAPMAN, J.D., FRANKO, A.J. & SHARPLIN, J. (1981). A marker for hypoxic cells in tumours with potential clinical applicability. *Br. J. Cancer*, **43**, 546-550.

CHAPMAN, J.D., URTASUN, R.C., FRANKO, A.J., RALEIGH, J.A., MEEKER, B.E. & MCKINNON, S.A. (1989). The measurement of oxygenation status of individual tumors. In *Prediction of Response in Radiation Therapy: Part 1. the Physical and Biological Basis*. Paliwal, B.R., Fowler, J.F., Herbert, D.E., Kinsella, T.J. and Orton, C.G. (eds.) American Institute of Physics: New York pp. 49-60.

COBB, L.M., HACKER, T. & NOLAN, J. (1990*a*). NAD(P)H nitroblue tetrazolium reductase levels in apparently normoxic tissues: A histochemical study correlating enzyme activity with binding of radiolabelled misonidazole. *Br. J. Cancer*, **61**, 524-529.

COBB, L.M., NOLAN, J. & BUTLER, S. (1990*b*). Tissue distribution of $^{14}$C- and $^{3}$H-labelled misonidazole in the tumor-bearing mouse. *Int. J. Radiat. Oncol. Biol. Phys.*, **18**, 347-351.

COBB, L.M., NOLAN, J. & BUTLER, S.A. (1990*c*). Distribution of pimonidazole and RSU 1069 in tumour and normal tissues. *Br. J. Cancer*, **62**, 915-918.

COBB, L.M., NOLAN, J. & O'NEILL, P. (1989). Microscopic distribution of misonidazole in mouse tissues. *Br. J. Cancer*, **59**, 12-16.

COLEMAN, C.N., URTASUN, R.C., WASSERMAN, T.H., HANCOCK, S., HARRIS, J., HALSEY, J. & HIRST, V.K. (1984). Initial report of the phase I trial of the hypoxic cell radiosensitizer SR 2508. *Int. J. Radiat. Oncol. Biol. Phys.*, **10**, 1749-1753.

ERNSTER, L. (1967). DT-diaphorase. *Methods Enzymol.*, **10**, 309-317.

FRANKO, A.J. & CHAPMAN, J.D. (1982). Binding of $^{14}$C-misonidazole to hypoxic cells in V79 spheroids. *Br. J. Cancer*, **45**, 694-699.

FRANKO, A.J. (1986). Misonidazole and other hypoxia markers: Metabolism and applications. *Int. J. Radiat. Oncol. Biol. Phys.*, **12**, 1195-1202.

FRANKO, A.J., KOCH, C.J., GARRECHT, B.M., SHARPLIN, J. & HUGHES, D. (1987). Oxygen dependence of binding of misonidazole to rodent and human tumors *in vitro*. *Cancer Res.*, **47**, 5367-5376.

GARRECHT, B.M. & CHAPMAN, J.D. (1983). The labelling of EMT-6 tumours in Balb/C mice with 14C-misonidazole. *Br. J. Radiol.*, **56**, 745-753.

GUICHARD, M. (1991). The use of fluorocarbon emulsions in cancer radiotherapy. *Radiother. Oncol. Suppl.*, **20**, 59-64.

HORSMAN, M.R., WOOD, P.J., CHAPLIN, D.J., BROWN, J.M. & OVERGAARD, J. (1990). The potentiation of radiation damage by nicotinamide in the SCC VII tumour *in vivo*. *Radiother. Oncol.*, **18**, 49-57.

KEYES, S.R., ROCKWELL, S. & SARTORELLI, A.C. (1985). Enhancement of mitomycin C cytotoxicity to hypoxic tumor cells by dicoumarol *in vivo* and *in vitro*. *Cancer Res.*, **45**, 213-216.

KOCH, C.J. (1990). The reductive activation of nitroimidazoles: Modification by oxygen and other redox-activated molecules in cellular systems. In *Selective Activation of Drugs by Redox Processes*. Adams, G.E., Breccia, A., Fielden, E.M. and Wardman, P. (eds.), Plenum Press; London, pp 237-247.

KOCH, C.J., STOBBE, C.C. & BAER, K. (1984). Metabolism induced binding of $^{14}$C-misonidazole to hypoxic cells: Kinetic dependence on oxygen concentration and misonidazole concentration. *Int. J. Radiat. Oncol. Biol. Phys.*, **10**, 1327-1331.

MACMANUS, M.P., MAXWELL, A.P., ABRAM, W.P. & BRIDGES, J.M. (1989). The effect of hypobaric hypoxia on misonidazole binding in normal and tumour-bearing mice. *Br. J. Cancer*, **59**, 349-352.

MANNAN, R.H., SOMAYAJI, V.V., LEE, J., MERCER, J.R., CHAPMAN, J.D. & WIEBE, L.I. (1991). Radioiodinated 1-(5-iodo-5′-deoxy-D-arabinofuranosyl)-2-nitroimidazole (Iodoazomycin arabinoside: IAZA): A novel marker of tissue hypoxia. *J. Nucl. Med.*, **32**, 1764-1770.

MARSHALL, R.S., PATERSON, M.C. & RAUTH, A.M. (1989). Deficient activation by a human cell strain leads to mitomycin resistance under aerobic but not hypoxic conditions. *Br. J. Cancer*, **59**, 341-346.

MAXWELL, A.P., MACMANUS, M.P. & GARDINER, T.A. (1989). Misonidazole binding in murine liver tissue: a marker of cellular hypoxia *in vivo*. *Gastroenterology*, **97**, 1300-1303.

OLIVE, P.L., RASEY, J.S. & DURAND, R.E. (1986). Comparison between the binding of [$^{3}$H] misonidazole and AF-2 in Chinese hamster V79 spheroids. *Radiat. Res.*, **105**, 105-114.

OVERGAARD, J., SAND HANSEN, H., LINDELOV, B., OVERGAARD, M., JORGENSEN, K., RASMUSSON, B. & BERTHELSEN, A. (1991). Nimorazole as a hypoxic radiosensitizer in the treatment of supraglottic larynx and pharynx carcinoma. First report from the Danish Head and Neck Cancer Study (DAHANCA) protocol 5-85. *Radiother. Oncol. Suppl.*, **20**, 143-149.

PARLIAMENT, M.B., CHAPMAN, J.D., URTASUN, R.C., MCEWAN, A.J., GOLBERG, L., MERCER, J.R., MANNAN, R.H. & WIEBE, L.I. (1992). Non-invasive assessment of human tumour hypoxia with $^{123}$I-iodoazomycin arabinoside: preliminary report of a clinical study. *Br. J. Cancer*, **65**, 90-95.

RALEIGH, J.A., FRANKO, A.J., TREIBER, E.O., LUNT, J.A. & ALLEN, P.S. (1986). Covalent binding of fluorinated 2-nitroimidazole to EMT-6 tumors in BALB/c mice: detection by F-19 nuclear magnetic resonance at 2.35 T. *Int. J. Radiat. Oncol. Biol. Phys.*, **12**, 1243-1245.

RASEY, J.A., KOH, W.J., GRIERSON, J.R., GRUNBAUM, Z. & KROHN, K.A. (1989) Radiolabelled fluoromisonidazole as an imaging agent for tumor hypoxia. *Int. J. Radiat. Oncol. Biol. Phys.*, **17**, 985-991.

RAUTH, A.M. (1984). Pharmacology and toxicology of sensitizers: Mechanism studies. *Int. J. Radiat. Oncol. Biol. Phys.*, **10**, 1293-1300.

TEICHER, B.A. & ROSE, C.M. (1984). Oxygen-carrying perflurochemical emulsion as an adjuvant to radiation therapy in mice. *Cancer Res.*, **44**, 4285-4288.

TWENTYMAN, P.R., BROWN, J.M., GRAY, J.W., FRANKO, A.J., SCOLES, M.A. & KALLMAN, R.F. (1980). A new mouse tumor model system (RIF-1) for comparison of end-point studies. *J. Natl. Cancer Inst.*, **64**, 595-604.

URTASUN, R.C., KOCH, C.J., FRANKO, A.J., RALEIGH, J.A. & CHAPMAN, J.D. (1986). A novel technique for measuring human tissue $pO_2$ at the cellular level. *Br. J. Cancer*, **54**, 453-457.

VAN OS-CORBY, D.J. & CHAPMAN, J.D. (1986). *In vitro* binding of $^{14}$C-misonidazole to hepatocytes and hepatoma cells. *Int. J. Radiat. Oncol. Biol. Phys.*, **12**, 1251-1254.

VAN OS-CORBY, D.J. & CHAPMAN, J.D. (1987). Is misonidazole binding to mouse tissues a measure of cellular $pO_2$? *Biochem. Pharmacol.*, **36**, 3487-3494.

WASSERMAN, T.H., LEE, D.J., COSMATOS, D., COLEMAN, C.N., PHILLIPS, T., DAVIS, L., MARCIAL, V. & STETZ, J. (1991). Clinical trials with etanidazole (SR-2508) by the Radiation Therapy Oncology Group (RTOG). *Radiother. Oncol. Suppl.*, **20**, 129-135.

WORKMAN, P., WALTON, M.I., POWIS, G. & SCHLAGER, J.J. (1989). DT-diaphorase: questionable role in mitomycin-C resistance, but a target for novel bioreductive drugs? (Letter). *Br. J. Cancer*, **60**, 800-802.